# Seismic Assessment of Footbridges under Spatial Variation of Earthquake Ground Motion (SVEGM): Experimental Testing and Finite Element Analyses

**DOI:** 10.3390/s20041227

**Published:** 2020-02-24

**Authors:** Izabela Joanna Drygala, Joanna Maria Dulinska, Maria Anna Polak

**Affiliations:** 1 Cracow University of Technology, Faculty of Civil Engineering, 31-155 Cracow, Poland; jdulinsk@pk.edu.pl; 2 University of Waterloo, Department of Civil and Environmental Engineering, Waterloo, ON N2L 3G1, Canada; polak@uwaterloo.ca

**Keywords:** spatial variation of earthquake ground motion, non-destructive testing, footbridges, seismic assessment, nonuniform ground excitations, seismic assessment

## Abstract

In this paper, the seismic assessments of two footbridges, i.e., a single-span steel frame footbridge and a three-span cable-stayed structure, to the spatial variation of earthquake ground motion (SVEGM) are presented. A model of nonuniform kinematic excitation was used for the dynamic analyses of the footbridges. The influence of SVEGM on the dynamic performance of structures was assessed on both experimental and numerical ways. The comprehensive tests were planned and carried out on both structures. The investigation was divided into two parts: in situ experiment and numerical analyses. The first experimental part served for the validation of both the finite element (FE) modal models of structures and the theoretical model of nonuniform excitation as well as the appropriateness of the FE procedures used for dynamic analyses. First, the modal properties were validated. The differences between the numerical and the experimental natural frequencies, obtained using the operational modal analysis, were less than 10%. The comparison of the experimental and numerical mode shapes also proved a good agreement since the modal assurance criterion values were satisfactory for both structures. Secondly, nonuniform kinematic excitation was experimentally imposed using vibroseis tests. The apparent wave velocities, evaluated from the cross-correlation functions of the acceleration-time histories registered at two consecutive structures supports, equaled 203 and 214 m/s for both structures, respectively. Also, the coherence functions proved the similarity of the signals, especially for the frequency range 5 to 15 Hz. Then, artificial kinematic excitation was generated on the basis of the adopted model of nonuniform excitation. The obtained power spectral density functions of acceleration-time histories registered at all supports as well as the cross-spectral density functions between registered and artificial acceleration-time histories confirmed the strong similarity of the measured and artificial signals. Finally, the experimental and numerical assessments of the footbridges performance under the known dynamic excitation generated by the vibroseis were carried out. The FE models and procedures were positively validated by linking full-scale tests and numerical calculations. In the numerical part of the research, seismic analyses of the footbridges were conducted. The dynamic responses of structures to a representative seismic shock were calculated. Both the uniform and nonuniform models of excitation were applied to demonstrate and quantify the influence of SVEGM on the seismic assessment of footbridges. It occurred that SVEGM may generate non-conservative results in comparison with classic uniform seismic excitation. For the stiff steel frame footbridge the maximum dynamic response was obtained for the model of nonuniform excitation with the lowest wave velocity. Especially zones located closely to stiff frame nodes were significantly more disturbed. For the flexible cable-stayed footbridge, in case of nonuniform excitation, the dynamic response was enhanced only at the points located in the extreme spans and in the midspan closely to the pillars.

## 1. Introduction

Dynamic properties of footbridges have been extensively studied in recent decades. Especially, improvements in the behavior of structures, with regard to their susceptibility to a variety of human-induced dynamic impacts, were examined [1,2]. Other considerations, like aerodynamic behavior, correlation of numerical results with measured dynamic properties, and seismic assessment [3,4,5,6,7] were also investigated. After the earthquake in New Zealand in September 2010, severe damages of footbridges were observed [8]. The Medway St footbridge in Christchurch became a symbol of the destructive power of the September 2010 earthquake.

In seismic assessment, it is reasonable to consider the spatial variation of earthquake ground motion (SVEGM) for long footbridges, which dimensions are comparable with lengths of seismic waves. Like other so-called multiple-support structures, they are exposed to SVEGM [9,10,11,12,13] and their foundations undergo concurrently various kinematic excitation in terms of amplitudes and frequencies.

The influence of SVEGM on the dynamic performance of bridges has been widely studied since the data collected from seismic arrays (e.g., SMART 1 strong motion array in Taiwan) became available [13]. The following factors causing SVEGM were recognized; wave passage effect (time delay of excitation at various points of a structure foundation), incoherence effect (loss of coherence resulting from wave reflection and refraction in a nonuniform foundation ground), and local soil/site effect (difference in ground conditions in particular points of the ground) [11]. In general, researchers have proved that the SVEGM effects tend to decrease the global structural response to seismic shocks. However, the significant pseudo-static component of structural response, increasing of the global response, was also recognized [11]. This effect was observed for the higher, antisymmetric, modes of vibration excitations [14,15]. Léger et al. [16] compared the results from analysis of the four-span bridge where the wave velocity was varied. The comparison showed that the dynamic response increases as the wave velocity decreases. Zembaty [17] provides the numerical investigation of a four-span bridge and discusses the random vibrations of a structure kinematic wave excitations. Sextos et al. [18] explored the results from the in situ monitoring of the Evripos bridge. The work focused on the analysis of the SVEGM effect during two earthquakes and on the dynamic performance of the structure. The authors documented, based on in situ test, that the asynchronous kinematic loading of the structure excited higher modes while reducing the vibrations on its fundamental natural frequency. The seismic assessments based on concept of SVEGM were also presented for other multiple-support structures, like dams, pipelines, or tunnels [19,20,21].

Different models of nonuniform of kinematic excitation were implemented to represent SVEGM. The basic models consider only wave passage effect [16]. More developed models takes into account incoherence effect [22]. The most advanced models are formulated considering also local site effects [11,23]. Spatial variability of earthquakes is also considered in EC 8 standard [24]. For the design purposes, this effect is taken into account for bridges at least 600 m long or when the geological discontinues are observed. The EC 8 also provides the set of models and methods of seismic assessment of structures to SVEGM. 

The main objective of this research was to explore the influence of SVEGM on seismic assessment of two pedestrian bridges on both experimental and numerical ways. A single-span steel frame and a three-span cable-stayed footbridge were taken into consideration. The comprehensive experimental testing was planned and carried out on both structures. 

Results of numerical analyses gain reliability when they undergo positive experimental validation. Otherwise, without insightful experimental control, it is difficult to state with certainty that the outcomes resulted from the FE modeling and procedures are an accurate representation of structural performance. This was the concept behind the authors’ framework for linking in situ tests and numerical assessment of seismic behavior of the footbridges. 

The investigation was divided into two parts: in situ tests and numerical seismic assessment of the footbridges. The first experimental part served for the comprehensive validation of both the FE models of structures and the adopted theoretical model of nonuniform excitation. Moreover, the dynamic responses of footbridges to known kinematic excitations were recognized in both experimental and numerical ways. The comparison of the measurements and calculations allowed for the global validation of the FE models as well as procedures used in dynamic calculations. Once the comprehensive experimental validation was provided, the seismic assessment of the footbridges under a selected seismic shock was carried out in the second part of the research. The analyses allowed demonstration and quantification of the influence of SVEGM on the seismic assessment of footbridges. Especially, the performed calculations enabled to recognize whether the SVEGM phenomenon may generate non-conservative results in comparison with results of classic analysis in which uniform seismic excitation is assumed.

The main novelty of the presented research lies in performing experimental studies on real footbridges together with confronting the obtained experimental results with the results of numerical simulations. The presented in situ validation of numerical results concerns three aspects: the modal properties of two real footbridges, the theoretical model of nonuniform kinematic excitation and the dynamic behavior of the structures under the known excitation through the comparison of the measured and calculated dynamic responses of both footbridges. Especially, the experimental in situ validation of the model of nonuniform kinematic excitation as well as the experimental check-up of the structures’ responses to nonuniform excitation make this study innovative in the field of civil engineering. These aspects are not highlighted in other studies.

## 2. Theoretical Background of Spatial Variation of Earthquake Ground Motion

In the paper, numerical approach was applied for the dynamic analysis of the footbridges. The dynamic responses of structures to kinematic excitations were evaluated by the time history analysis using direct integration method for the solution of equations of motion. The mathematical formulation of motion of a general multi-degree of freedom structure under kinematic loading takes the following form [11,25],

(1) $$\left[\begin{array}{cc} M_{ss} & M_{sg}\\ M_{gs} & M_{gg} \end{array}\right]\left\{\begin{array}{c} {\ddot{u}}_{s}^{t}\\ {\ddot{u}}_{g} \end{array}\right\}+\left[\begin{array}{cc} C_{ss} & C_{sg}\\ C_{gs} & C_{gg} \end{array}\right]\left\{\begin{array}{c} {\dot{u}}_{s}^{t}\\ {\dot{u}}_{g} \end{array}\right\}+\left[\begin{array}{cc} K_{ss} & K_{sg}\\ K_{gs} & K_{gg} \end{array}\right]\left\{\begin{array}{c} u_{s}^{t}\\ u_{g} \end{array}\right\}\;=\;\left\{\begin{array}{c} 0\\ F_{g} \end{array}\right\},$$

where s—degrees of freedom of the structure; g—degrees of freedom of the ground; [M]—a mass matrix; [C]—a damping matrix; [K]—a stiffness matrix; $\left\{{\ddot{u}}_{s}^{t}\right\}$, $\left\{{\dot{u}}_{s}^{t}\right\}$, and $\left\{u_{s}^{t}\right\}$—vectors of total accelerations, velocities, and displacements of the structure, respectively; $\left\{{\ddot{u}}_{g}\right\},$ $\left\{{\dot{u}}_{g}\right\}$, and $\left\{u_{g}\right\}$—vectors of total accelerations, velocities and displacements of the ground motion, respectively; $\left\{F_{g}\right\}$—a vector of reactions at the support degrees of freedom.

Matrix Equation (1) can be converted into two separate equations. The first equation is given by Formula (2):

(2) $$\left[M_{ss}\right]\left\{{\ddot{u}}_{s}^{t}\right\}+\left[C_{ss}\right]\left\{{\dot{u}}_{s}^{t}\right\}+\left[K_{ss}\right]\left\{u_{s}^{t}\right\}\;=\;-\left[M_{sg}\right]\left\{{\ddot{u}}_{g}\right\}-\left[C_{sg}\right]\left\{{\dot{u}}_{g}\right\}-\left[K_{sg}\right]\left\{u_{g}\right\}.$$

The second equation resulting from conversion of Equation (1) is necessary only for calculation of support forces occurring due to the kinematic excitation. It is omitted in the paper.

The vector of total displacements of the structure $\left\{u_{s}^{t}\right\}$ (as well as vectors of total velocities and accelerations) consists of two parts, i.e., $\left\{u_{s}^{d}\right\}$—the dynamic component and $\left\{u_{s}^{p}\right\}$—the quasi-static component [11,25]. Therefore, the vector of total displacements can be quantified as follows.

(3) $$\left\{u^{t}\right\}\;=\;\left\{\begin{array}{c} u_{s}^{d}\\ 0 \end{array}\right\}+\left\{\begin{array}{c} u_{s}^{p}\\ u_{g} \end{array}\right\}.$$

The quasi-static component is expressed by Equation (4):

(4) $$\left\{u_{s}^{p}\right\}\;=\;\left[R\right]\left\{u_{g}\right\},$$

where [R]—a transformation matrix, which is expressed with the formula

(5) $$\left[R\right]=\;-\;\left[K_{ss}^{-1}\right]\left[K_{sg}\right].$$

After including Equations (3)–(5), Equation (2) becomes equivalent to

(6) $$\left[M_{ss}\right]\left\{{\ddot{u}}_{s}^{d}\right\}+\left[C_{ss}\right]\left\{{\dot{u}}_{s}^{d}\right\}+\left[K_{ss}\right]\left\{u_{s}^{d}\right\}=\;\left(\left[M_{ss}\right]\left[K_{ss}^{-1}\right]\left[K_{sg}\right]-\left[M_{sg}\right]\right)\left\{{\ddot{u}}_{g}\right\}+\left(\left[C_{ss}\right]\left[K_{ss}^{-1}\right]\left[K_{sg}\right]-\left[C_{sg}\right]\right)\left\{{\dot{u}}_{g}\right\}.$$

EC 8 [24] allows for skipping the second element of the right hand side of Equation (6), especially in the case of the Rayleigh stiffness-proportional model of damping. Taking into account Formula (5), which describes the transformation matrix [R], the following equation of motion of a structure under kinematic excitation can be formulated,

(7) $$\left[M_{ss}\right]\left\{{\ddot{u}}_{s}^{d}\right\}+\left[C_{ss}\right]\left\{{\dot{u}}_{s}^{d}\right\}+\left[K_{ss}\right]\left\{u_{s}^{d}\right\}=\;\left(-\left[M_{ss}\right]\left[R\right]-\left[M_{sg}\right]\right)\left\{{\ddot{u}}_{g}\right\}.$$

The dynamic response of a structure to kinematic excitation obtained by numerical integration of Equation (7) depends on the ground accelerations vector $\left\{{\ddot{u}}_{g}\right\}$. The individual components of this vector represent time histories of the ground accelerations at particular supports of the structure. However, time histories of accelerations are usually registered by a seismological station at one control point only. Therefore, if nonuniform kinematic excitation is intended in numerical simulations of a multiple-support structure, application of Equation (7) requires additionally an assumption of a model of excitation. 

In this study a model of nonuniform kinematic excitation taking into consideration only the wave passage effect was adopted. In the model it is assumed that subsequent points of the ground in the direction of wave propagation repeat the same motions with a time delay dependent on wave velocity. Therefore, the appropriate assumption of wave velocity is of a crucial meaning for dynamic calculations of a multiple-support structure [9,12,26].

## 3. General Framework of the Research

**Figure 1:**
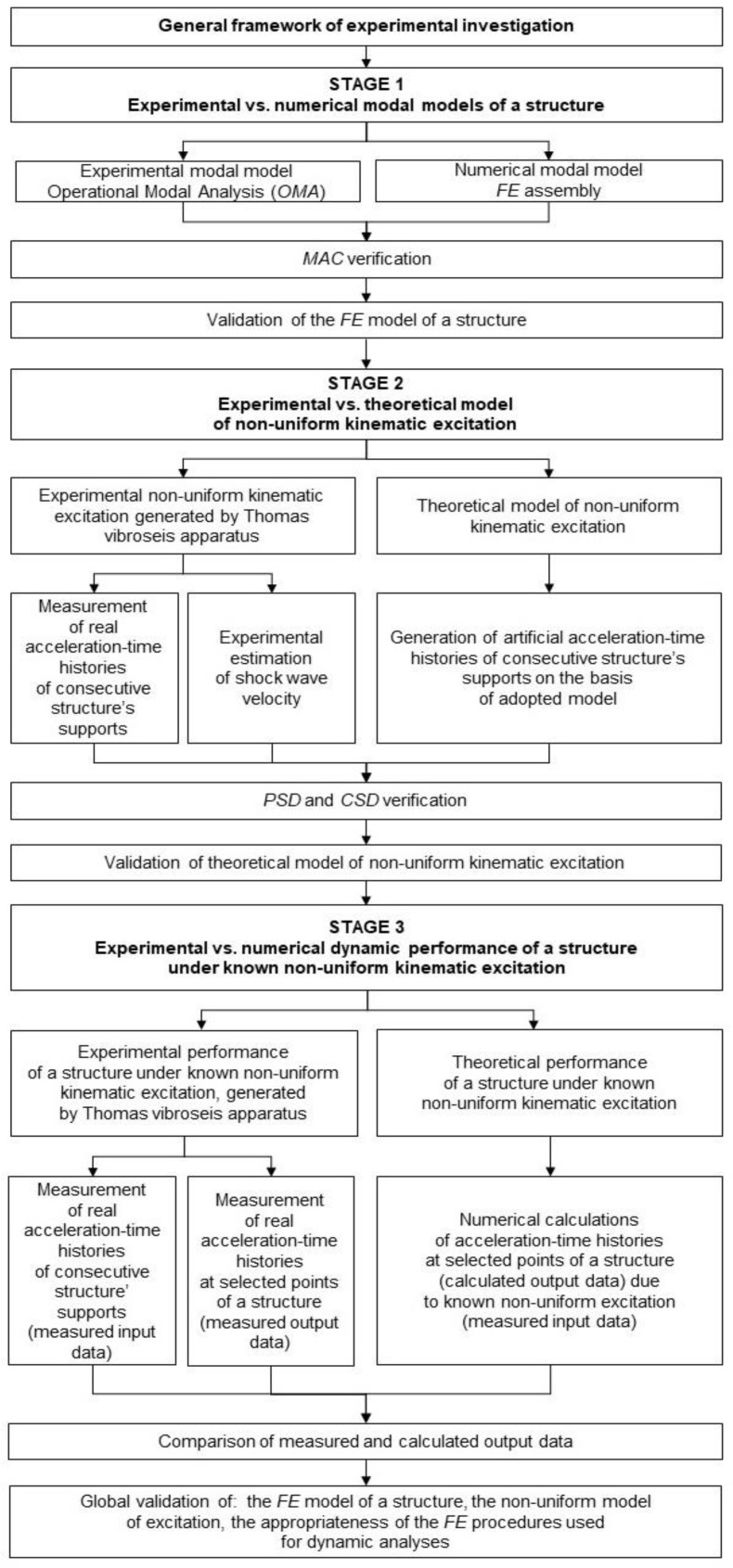
General concept of the experimental investigation carried out in three stages: 1st stage—the validation of the modal models of both footbridges with regard to the results of in situ experiment, 2nd stage—the validation of the theoretical model of nonuniform kinematic excitation through the similarity assessment of the measured and artificial excitations, and 3rd stage—the validation of the dynamic responses of the footbridges through the comparison of the measured and calculated accelerations at representative points of the structure.

The research was divided into experimental, in situ, and numerical parts. The general framework of the in situ tests is shown in Figure 1. Identical framework was executed for both analyzed footbridges. This part of the research was carried out in three stages.

The first stage of in situ investigation allowed for the validation of the FE models of both footbridges. In this stage, the following steps were conducted (see Figure 1).

- The experimental modal models of both footbridges were created employing the operational modal analysis (OMA) techniques.
- The numerical modal models of both footbridges were obtained through assembling the FE models of both footbridges and the calculation of dynamic properties of both structures.
- The validation of the FE models of both footbridges with regard to the results of in situ experiment was carried out using the modal assurance criterion (MAC) theory.

The second stage of the in situ investigation enabled the experimental validation of the adopted theoretical model of nonuniform kinematic excitation. In this stage, the following steps were carried out (see Figure 1).

- The experimental measurements of bridges’ responses resulting from nonuniform kinematic excitation generated by the vibroseis, i.e., measurements of acceleration-time histories on the consecutive structural supports and estimation of shock wave velocities on the basis of the cross-correlation (CCr) functions of the acceleration-time histories registered at two consecutive structures’ supports.
- The generation of artificial acceleration-time histories for the consecutive supports of both footbridges, based on the adopted model of nonuniform kinematic excitation.
- The validation of the theoretical model of nonuniform kinematic excitation through the similarity assessment of the measured and artificial excitations using the power spectral density (PSD) and the cross-spectral density (CSD) functions.

The third stage of in situ investigation enabled the validation of dynamic performance of the footbridges as well as the appropriateness of the FE procedures used for dynamic analyses. In this stage, the following steps were carried out (see Figure 1).

- The experimental measurements of the dynamic responses of both footbridges (in terms of acceleration-time histories registered at selected output measurement points of the structures) under nonuniform excitation of known amplitudes and frequency range, generated by the vibroseis placed in close proximity to a footbridge.
- The numerical recognition of the dynamic responses of footbridges (in terms of acceleration-time histories calculated at selected output measurement points of the structures) under known nonuniform excitation generated by the vibroseis.
- The validation of both the FE models of both footbridges and the nonuniform model of excitation as well as the FE procedures used for dynamic analyses through the comparison of the measured and calculated dynamic responses of both footbridges.

In the numerical part of the research the seismic analyses of the footbridges were conducted. The dynamic responses of the structures to a selected seismic shock were calculated. Both the uniform and nonuniform models of kinematic excitation were applied for the dynamic calculations that enabled to demonstrate and quantify the influence of the SVEGM phenomenon on the seismic assessments of both footbridges. Based on the comprehensive experimental validation of the FE models conducted in the first, in situ, part of the research, it can be concluded that the results of the numerical seismic analyses reflected the real performance of the footbridges subjected to the SVEGM phenomenon.

## 4. Structural Layouts and FE Models of the Footbridges

**Figure 2:**
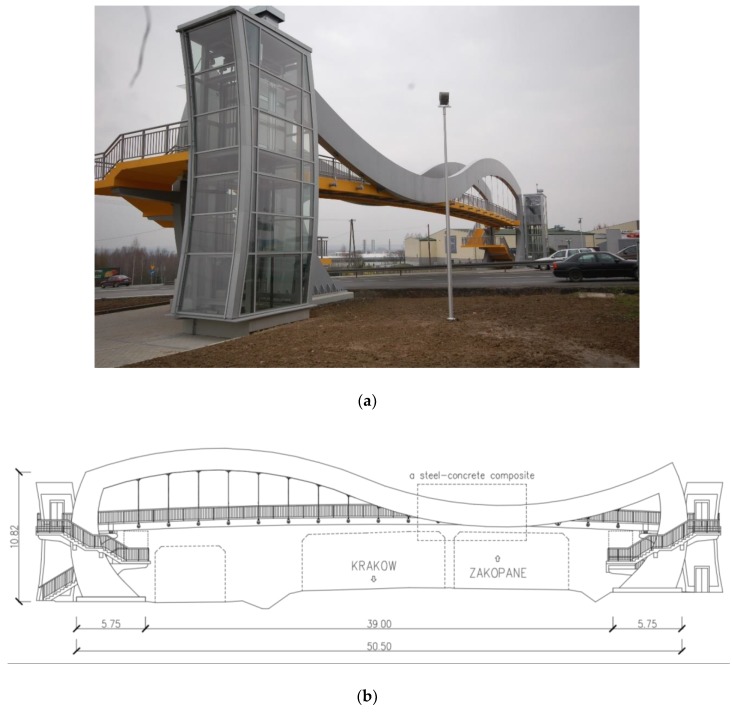
The single-span steel frame footbridge: (**a**) general view and (**b**) structural layout.

Two pedestrian bridges were selected for the investigation. The first footbridge is a single-span steel frame structure (Figure 2). The primary structural system consists of two steel frames, which are uniform box sections with 30.00 mm wall thickness. The footbridge deck is a steel-reinforced concrete slab supported by concrete girders and steel cross bars. The deck is attached to the steel frames with elastomeric bearings and steel hangers as linking elements. The parts of the steel frames are fully connected to the footbridge deck forming a steel–concrete composite. The total length of the steel frames is 50.50 m. They are fixed in the steel-reinforced concrete pile cups. The abutments are supported on reinforced concrete piles. The structure is relatively stiff in comparison with other typical footbridges.

**Figure 3:**
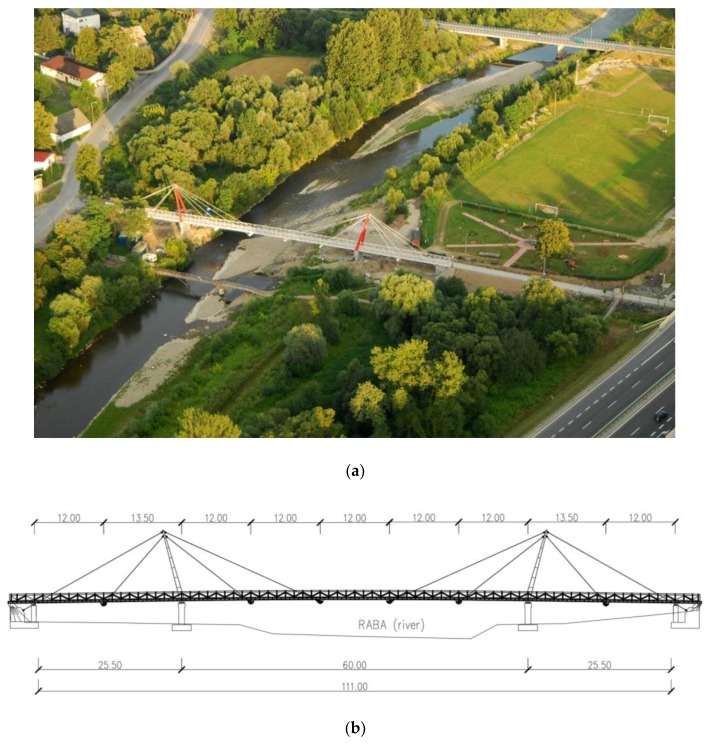
The cable-stayed footbridge: (**a**) general view and (**b**) structural layout.

The second footbridge is the three-span cable-stayed bridge (Figure 3). The suspended structure is composed of three spans—the central span is 60.00 m long and the two outer spans are 25.50 m long. The total length of the footbridge is 120.00 m. The footbridge deck is a composite of steel girders and a steel-reinforced concrete slab with a thickness of 15.00–18.00 cm. The depth, flange width, web thickness, and flange thickness of steel beams are the standard measurements of IPE360 (girders) and IPE220 (cross bars). The steel beams form a grid. The deck of the footbridge is connected to two steel pylons (11.80 m high) by cables. The structure is equipped with elastomeric bearings as linking elements between the deck and the abutments. The pillars and abutments are founded on reinforced concrete piles with a diameter of 100 cm. 

The 3D finite element (FE) models of both structures were assembled, and dynamic analyses were completed with the ABAQUS/Standard [27]. The structural parts of the frame bridge are represented by a deck and steel frames by shell elements, girders and cross bars by beam elements, abutments and elastomeric bearings by solid elements, and hangers by truss elements. The structural parts the cable-stayed footbridge are modeled by a deck, steel pylons, steel girders and steel cross bars by shell elements; abutments, pillars, and elastomeric bearings by solid elements; and cables by truss elements. 

The fixed boundary conditions, reflecting the high rigidity of the foundations as well as high stiffness of the subsoils, were applied at the end of the abutments and pillars. The following element types were used in the FE analyses (Abaqus referenced); linear quadrilateral shell elements of type S4R, quadratic hexahedral solid elements of type C3D20R, linear line beam elements of type B31, and elastic truss elements (T3D2) with no compressive stiffness (“no compression” option). The latter elements were used for modeling hangers and cables to guarantee that compressive stresses would not be generated during dynamic analysis [27]. However, when such numerical approach is used, instability of the model can appear. This difficulty was overcome by overlaying each truss element, which has no compression stiffness with beam element and has low compression stiffness. This enables obtaining a stiffness greater than zero, which has the effect of stabilizing the model. A level of 5% of the cables’ stiffness was selected for the stabilizing elements. “Tie” constraints were used to guarantee identical kinematic behavior of the truss and the beam elements. The modulus of elasticity of the steel elements was adopted as 210 GPa with the Poisson’s ratio of 0.29. Both structures are equipped with elastomeric bearings that are composed of two steel plates with elastomeric laminae in between. In the FE calculations, the homogenization theory was applied for the elastomeric laminae [28]. The geometrical parameters for the elastomeric bearings based on the real dimensions of bearings. Usually, a two-parameter Mooney–Rivlin model is used as a constitutive model of hyperelastic, nonlinear, elastomeric-bearing material. However, the parameters of the Mooney–Rivlin material (C10 and C01) can be replaced with the equivalent elasticity modulus: E = 6 (C10 + C01) [28]. In this paper, the parameters of the Mooney–Rivlin model (adopted as C10 = 0.292 MPa and C01 = 0.177 MPa [28]) were replaced with the equivalent elasticity modulus (2.814 MPa). The Poisson’s ratio of the elastomeric material was taken as 0.49. For the steel plates the elasticity modulus 210 GPa and Poisson’s ratio 0.3 were adopted.

## 5. Experimental Set-Up

**Figure 4:**
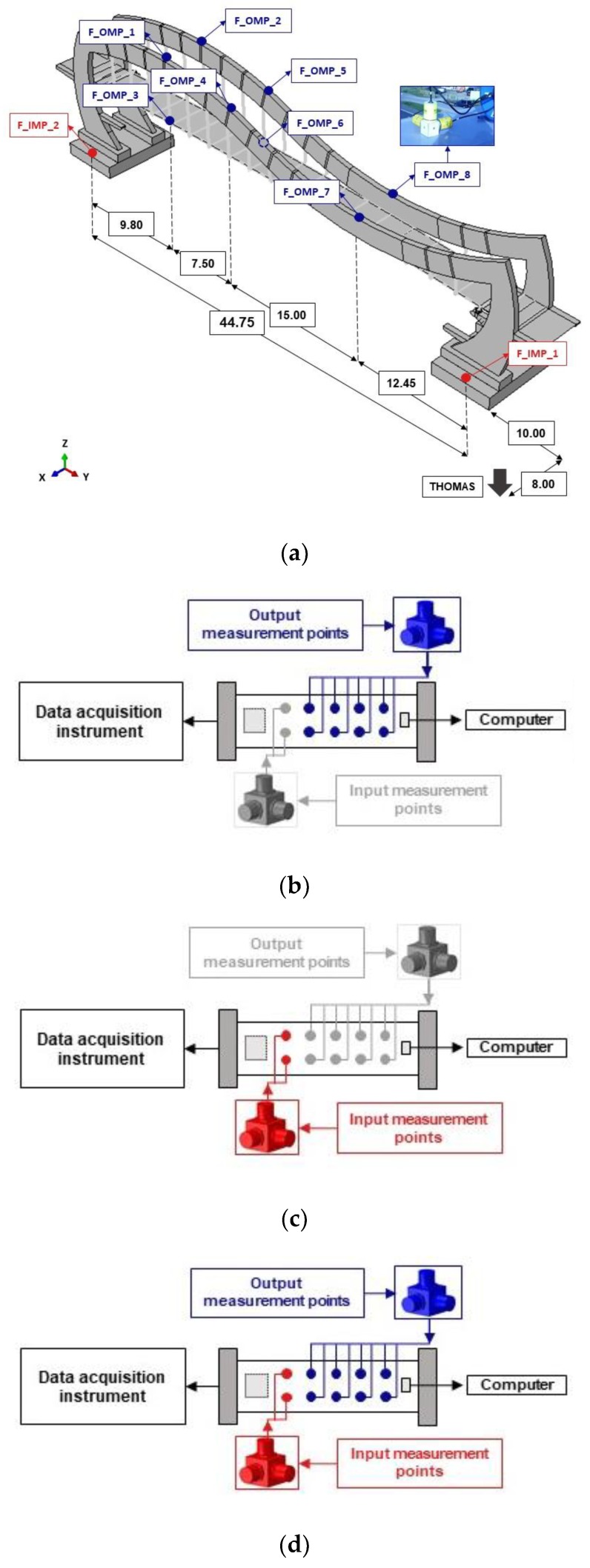
(**a**) Layout of measurement points and accelerometer anchorage for the frame footbridge (F_IMP—input measurement points; F_OMP—output measurement points); schemes of active measurement points for (**b**) the first, (**c**) second, and (**d**) third stage of the experiment.

**Figure 5:**
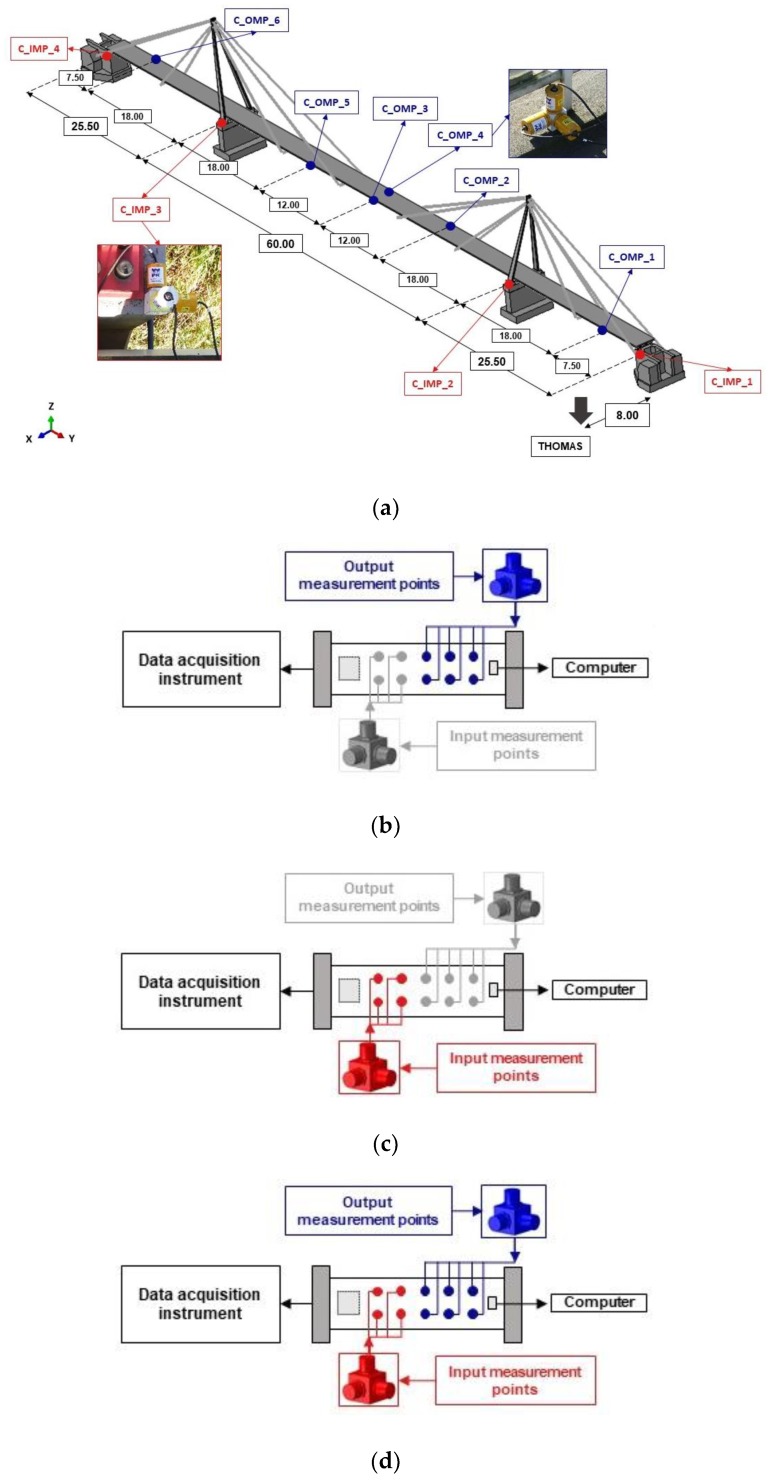
(**a**) Layout of measurement points and accelerometer anchorage for the cable-stayed footbridge (C_IMP—input measurement points; C_OMP—output measurement points); schemes of active measurement points for (**b**) the first, (**c**) second, and (**d**) third stage of the experiment.

The layouts of measurement points for the testing scenario realized for both footbridges are shown in Figure 4a and Figure 5a, respectively. The measurement points consisted of three piezoelectric high sensitivity (10,000 mV/g) accelerometers 393B12 PCB Piezotronics located in three directions. The frequency range of accelerometers was from 0.15 to 1000 Hz. All sensors were wire connected. Data sampling of the signal was 1024 Hz. 

During all tests the data were collected at input and output measurement points of both footbridges. The input measurement points—F_IMP for the frame (first bridge) and C_IMP for the cable-stayed footbridge (second bridge)—served for recording the motion of structures’ supports, whereas the output measurement points—F_OMP for the frame and C_OMP for the cable-stayed footbridge—were dedicated to register the motion of main elements of structural systems. 

In the first stage of the experimental part (see Figure 1), the validation of the FE modal models of the structures was performed using the operational modal analysis (OMA). For the OMA procedures only the output measurement points (F_OMP and C_OMP) were launched. Active measurement points, which served for data collection in this stage, are shown schematically in Figure 4b and Figure 5b for both footbridges, respectively.

In the second stage, the validation of the theoretical model of nonuniform kinematic excitation, adopted in this study, was carried out. For the experimental detection of nonuniform kinematic excitation, only the input measurement (F_IMP and C_IMP) were needed. Active measurement points, used for data collection in this stage, are presented in Figure 4c and Figure 5c. In the case of the frame footbridge the input points (F_IMP) were located on the structure’s foundation. As the footbridge foundation was rigid and the recorded stiffness of subsoil was high, accelerations registered on the foundation represented in fact the ground motion. In the case of the cable-stayed footbridge, the input points (C_IMP) were located on the top of each pillar of the structure. The high rigidity of foundation and pillars allowed considering the motion registered at pillars and the ground motion as being identical.

In the third stage, the global validation of the FE models of structures and the theoretical model of nonuniform excitation, as well as for the FE procedures used for dynamic analyses, was provided. For the global validation purposes both the input (F_IMP and C_IMP) as well as the output (F_OMP and C_OMP) measurement points had to be launched. Active measurement points, used for data collection in this stage, are presented in Figure 4d and Figure 5d.

**Figure 6:**
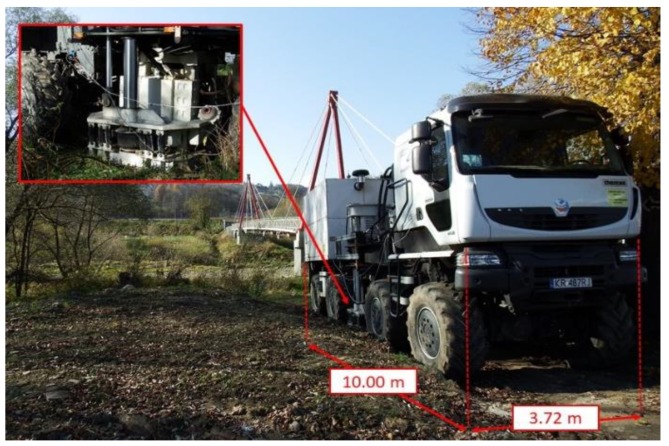
THOMAS vibroseis apparatus.

The nonuniform kinematic excitation ground vibrations were generated by the THOMAS vibroseis apparatus (see Figure 6). The vibroseis generates vibrations by a plate striking the ground. These vibrations are transmitted to foundations through the ground. The total mass of the apparatus is 32,000 kg and the range of excitation frequencies is 2–250 Hz. The locations of the vibroseis during the experiments are presented in Figure 4a and Figure 5a. These locations ensured that the input measurement points were placed along the direction of generated waves propagation. The vibroseis may execute sweeps, i.e., vibrations of frequency linearly or exponentially fluctuating in the time domain. 

**Figure 7:**
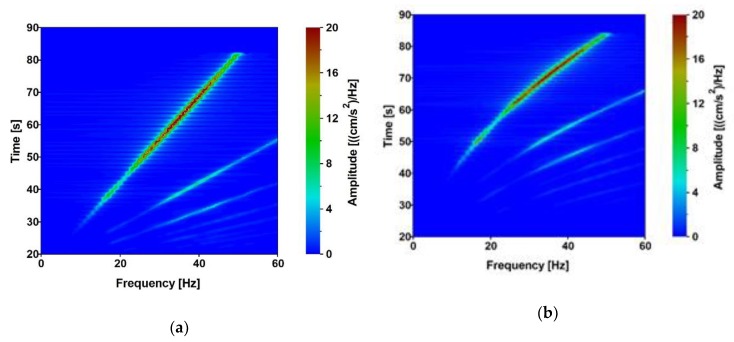
Time-frequency characteristics of the vibroseis tests: (**a**) linear sweep and (**b**) exponential sweep.

The time-frequency characteristics of both the linear and exponential sweeps are shown in Figure 7a,b, respectively. The data were transformed by the Short-Time Fourier Transform (STFT) with the rectangular window (13 dB W = 1) with normalization by amplitudes. The segment length of the STFT was 1664.

The time-frequency characteristics illustrate the intensity of the obtained signals and the time period of low frequencies registered at one footbridge support. It is visible in Figure 7 that better time-frequency characteristics (in terms of generating low frequencies with higher amplitudes) were obtained from the exponential sweep. Based on this observation, it was decided that the exponential sweeps will be generated for all further vibroseis tests.

## 6. Theoretical Background for In Situ Tests

A set of mathematical tools were applied for the analysis of signals [11,29]. The experimental estimation of dynamic properties of the footbridges was based on ambient vibrations caused by operational excitation. In the experiment, data at output measurement points were collected. As a natural frequency estimator of the experimental modal model, the summation of all combinations of auto- and cross-spectral density functions (PSD and CSD) between data recorded at all output measurement points was used. The peak picking method was used as the method for the estimation of natural frequency values. The frequencies for which extreme values of the estimator appeared were identified as being the eigenfrequencies of the structure. The auto-spectral density function is provided by Equation (8):

(8) $$S_{xx}\left(f\right)\;=\;\int_{-\infty }^{\infty }R_{xx}\left(\tau \right)e^{-j2\pi ft}dt,$$

where $R_{xx}$ is the autocorrelation for the x(t) signal registered in the control point. The autocorrelation function is given by the Formula (9):

(9) $$R_{xx}\left(\tau \right)\;=\;\underset{T\to \infty }{\lim}\frac{1}{T}\int_{0}^{T}x\left(t\right)x\left(t+\tau \right)dt$$

where t is time, x(t) is the data registered in the first control point, and τ is time delay. 

The cross-spectral density function is defined by Equation (10):

(10) $$S_{xy}\left(f\right)\;=\;\int_{-\infty }^{\infty }R_{xy}\left(\tau \right)e^{-j2\pi ft}dt,$$

where $R_{xy}$ is the cross-correlation for the x(t) and y(t) signals registered in the control point. The cross-correlation function is given by the Formula (11):

(11) $$R_{xy}\left(\tau \right)=\underset{T\to \infty }{\lim}\frac{1}{T}\int_{0}^{T}x\left(t\right)y\left(t+\tau \right)dt$$

where t is time, x(t) is data registered in the first control point, y(t) is data registered in the second control point, and τ is time of delay between x(t) and y(t) signals. The cross-correlation function has the maximum value $R_{xy}\left(\tau_{0}\right)$ for the $\tau_{0}$ that represents the time delay of the signal. 

The modal assurance criterion (MAC) was used as a mathematical tool for the verification of the obtained mode shapes from both modal models [29]. The MAC(i,j) values for the i and j eigenvectors were extracted on the basis of Equation (12):

(12) $$MAC\left(i,j\right)\;=\frac{{\left({\left\{\Psi_{i}\right\}}^{T}\left\{\Psi_{j}\right\}\right)}^{2}}{\left({\left\{\Psi_{i}\right\}}^{T}\left\{\Psi_{i}\right\}\right)\left({\left\{\Psi_{j}\right\}}^{T}\left\{\Psi_{j}\right\}\right)},$$

where $\Psi_{i}$, $\Psi_{j}$ are modal vectors. 

For duplicate mode shapes, the MAC(i,j) index has a value of 1; for different eigenvectors, the MAC(i,j) index has a value of 0. In practise, the boundaries of the MAC(i,j) values, which verify modal model positively, were quoted as being greater than 0.8 and less than 0.2. 

Spatial variability of earthquake ground motion means that the excitation at different points of a structure foundation is not identical. Due to the passage wave effect, the time delay in excitation registered at two supports of a structure appears. In the experiment, signals at the input measurement points of both structures were collected. The cross-correlation function (CCr), given by the formula (11), was used as an estimator that provides the time shift of a signal between two supports.

As the incoherence effect may significantly change the signals collected at different points, the frequency content of the signals that were registered in the input measurement points was investigated by the coherence function. The coherence function (CH) is a linear non-dimensional estimator, which is calculated from the signals x(t) and y(t):

(13) $$C_{xy}\left(f\right)\;=\;\frac{{\left(S_{xy}\left(f\right)\right)}^{2}}{S_{xx}\left(f\right)S_{yy}\left(f\right)},$$

where $S_{xx}\left(f\right)$ and $S_{yy}\left(f\right)$ are power spectra of signals x(t) and y(t), respectively; $S_{xy}\left(f\right)$ is cross-power spectrum for these signals; and f is frequency. 

The values of the $C_{xy}\left(f\right)$ are in range from 0 to 1. The value of coherence of 0 at a given frequency means there is no similarity between the data at this frequency. A coherence value of 1 at a given frequency means that the spectral contents at this frequency are identical [12]. 

In the signals generated by the vibroseis and registered on supports were compared with the numerical data generated based on the adopted model of nonuniform kinematic excitation. In this model, the decrease of vibration amplitudes, caused by distance from the source of vibration, was evaluated on the basis of Equation (14) [26]:

(14) $$A_{r}\;=\;A_{0}{\left(\frac{r_{0}}{r}\right)}^{n}e^{-\alpha \left(r-r_{0}\right)},$$

where $A_{r}$ is the amplitude of the vibration at distance r from the source of vibrations, $A_{0}$ is the measured amplitude of the vibration at distance $r_{0}$ from the source, n is the discrepancy factor (for the Rayleigh waves n = 0.5), and α is soil-dependent absorption factor (α = 0.01÷0.1).

## 7. Results of in Situ Experiments and Discussion

### 7.1. Stage 1: Experimental vs. Numerical Modal Models of the Structures

The experimental modal models of both footbridges were created using OMA techniques. The results of modal assessments for both structures were presented in details in works [6,7]. For the OMA procedures only the output measurement points (F_OMP and C_OMP) were launched (see Figure 4b and Figure 5b). 

**Figure 8:**
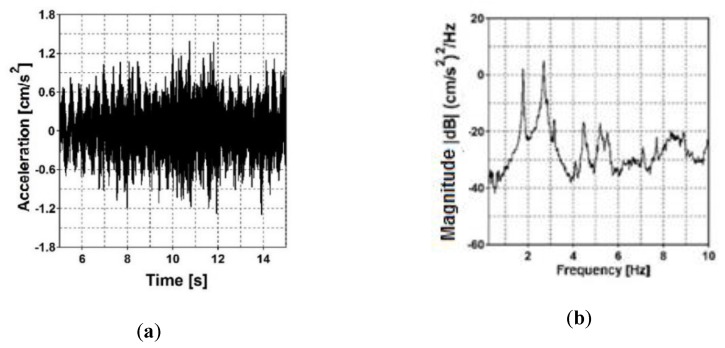
(**a**) Acceleration-time history at output measurement point F_OMP_2 of the frame footbridge and (**b**) natural frequency estimator.

In the case of the first frame footbridge, accelerations at two output points (F_OMA_1 and F_OMP_2) were registered. The 10 s fragment of acceleration-time history registered at point F_OMP_2 in the vertical direction as a result of ambient vibration is presented in Figure 8a. The natural frequency estimator of the experimental modal model (i.e., the summation of all combinations of PSD and CSD functions between data recorded at all output measurement points) is illustrated in Figure 8b. 

**Figure 9:**
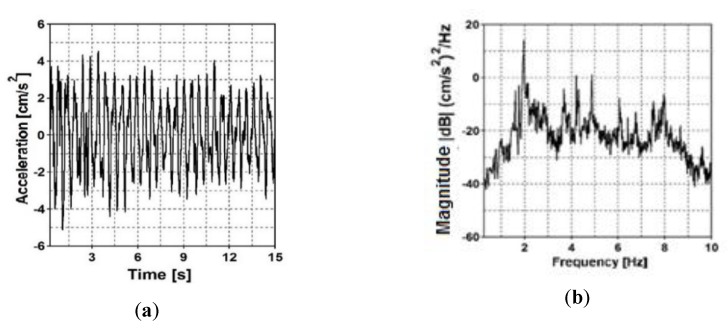
(**a**) Acceleration-time history at output measurement point C_OMP_3 of the cable-stayed footbridge and (**b**) natural frequency estimator.

In the case of the cable-stayed footbridge (second bridge), accelerations at six output points (form C_OMA_1 to C_OMP_6) were registered. The 15 s fragment of acceleration-time history registered at measurement point C_OMP_3 in the vertical direction as a result of ambient vibration is presented in Figure 9a. The natural frequency estimator of the experimental modal model is presented in Figure 9b. The natural frequencies of both footbridges estimated through the in situ investigation, are shown in Table 1, in which the experimental and numerical results and summarized.

The experimental estimation of modal shapes was conducted using ambient vibrations. The acceleration-time histories, registered at all output measurement points, were filtered around the consecutive natural frequencies of each footbridge. Third-order Butterworth band-pass filters with a width of 0.04 Hz were used in both cases. This filter is usually used for experimental modal shapes estimation as it provides (in the contrary to Chebyshev or elliptic filters) monotonic amplitude response without ripples in both passband and stopband as well as quick roll-off around the cutoff frequency [30]. Differences in phase and amplitudes of the filtered signals, registered by sensors placed at different points, indicated that they represented the natural modes accompanied with the obtained natural frequencies. 

The validation of the experimentally-obtained mode shapes was carried out by means of the AutoMAC tool [29]. On the basis of the AutoMAC values, which were less than 0.2 out of the diagonal for both footbridges, a correlation between mode shape vectors was ruled out. Therefore, sufficient number of the measurement points was installed to uniquely identify the individual modes.

**Figure 10:**
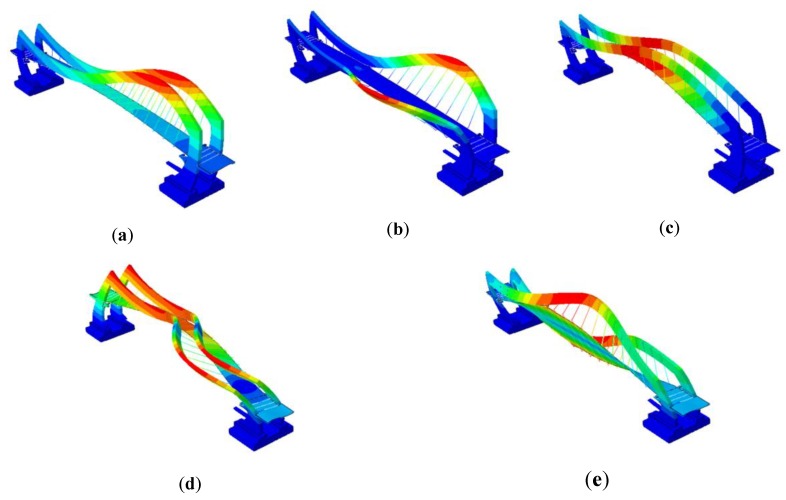
(**a**) 1st, (**b**) 2nd, (**c**) 3rd, (**d**) 4th, and (**e**) 5th mode shapes of the frame footbridge [6].

**Figure 11:**
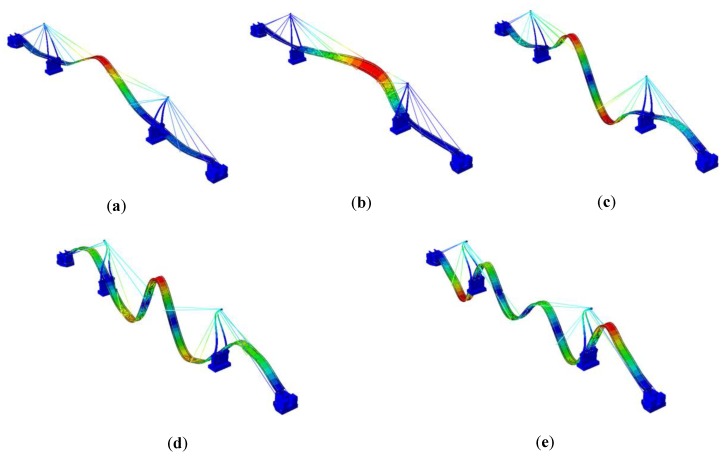
(**a**) 1st, (**b**) 2nd, (**c**) 3rd, (**d**) 4th, and (**e**) 5th mode shapes of the cable-stayed footbridge [7].

The numerical modal models of both footbridges were obtained through assembling the FE models of structures and the natural frequencies as well as mode shapes were estimated numerically (see Table 1). The mode shapes of both footbridges are shown in Figure 10 and Figure 11, respectively.

The *MAC* criterion was used to verify the numerical modes of both footbridges with respect to experimental results. The MAC indices related to different modes were not always equal zero, and the MAC indices related to the same modes were not always equal one. This inaccuracy may occur due to the limited number of output measurement points used in the in situ experiments. However, still more than 90% of the obtained MAC values fulfilled the criteria of being greater than 0.8 on the matrix diagonals and being less than 0.2 on the diagonals [29].

The logarithmic decrements of damping were also obtained experimentally for every estimated eigenvalue of both footbridges. The acceleration-time histories of all control points were filtered around the estimated natural frequencies. The free decay plots were obtained at every measurement point in all three directions. All obtained free decay plots enabled the estimation of average logarithmic decrements for all natural frequencies (see Table 1). 

The validation of the FE models of footbridges using the MAC theory was carried out. Experimentally obtained modal vectors provided data for verification of the FE modal models of the footbridges. The obtained results showed strong similarity as far as the natural frequencies and the values of the $MAC_{i,i}$ indices were taken into account. As a measure of the similarity, the errors between numerical and experimental results were evaluated. The errors are less than 10%, except for the fourth natural frequency of the frame footbridge. Again, this inaccuracy may have resulted from the limited number of output measurement points used in the in situ experiments of the frame footbridge. Therefore, the first stage of experimental investigation allowed for the positive validation of the FE models of the footbridges.

### 7.2. Stage 2: Experimental vs. Theoretical Model of Nonuniform Kinematic Excitation

The experimental detection of nonuniform kinematic excitation generated by the vibroseis, i.e., measurement of acceleration-time histories on the consecutive supports at input points (F_IMP and C_IMP) of the footbridges and estimation of shock wave velocities, was carried out. The ground vibrations were generated by the vibroseis (see Figure 6), placed in close proximity to the footbridges (see Figure 4a and Figure 5a). The exponential sweep with a frequency range of 2 to 50 Hz was realized. Total duration of the sweep was 60 s. In this stage only the input measurement points F_IMP and C_IMP were active (see Figure 4c and Figure 5c). 

**Figure 12:**
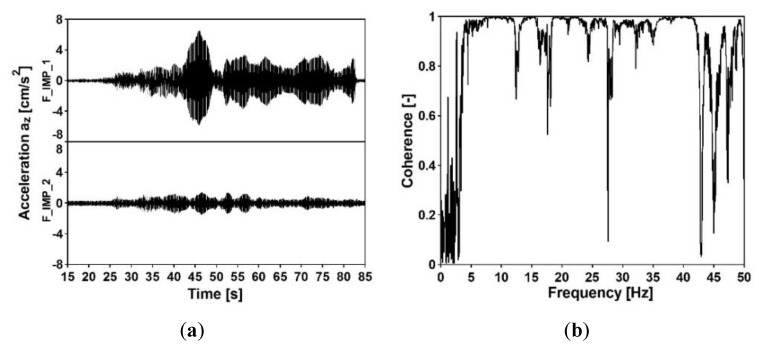
(**a**) Acceleration-time histories at input measurement points F_IMP_1 and F_IMP_2 of the frame footbridge due to the exponential sweep and (**b**) coherence function between the signals at points F_IMP_1 and F_IMP_2.

**Figure 13:**
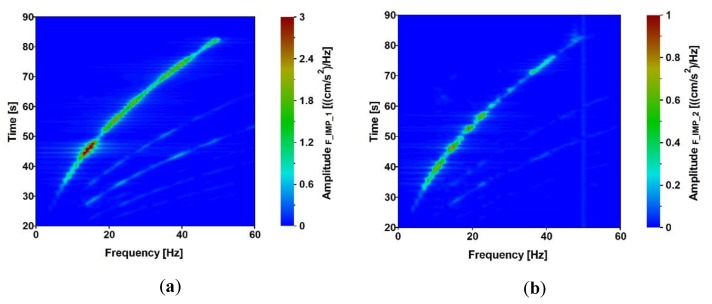
Time-frequency characteristics of the excitation at point (**a**) F_IMP_1 and (**b**) F_IMP_2 of the frame footbridge due to exponential sweep.

**Figure 14:**
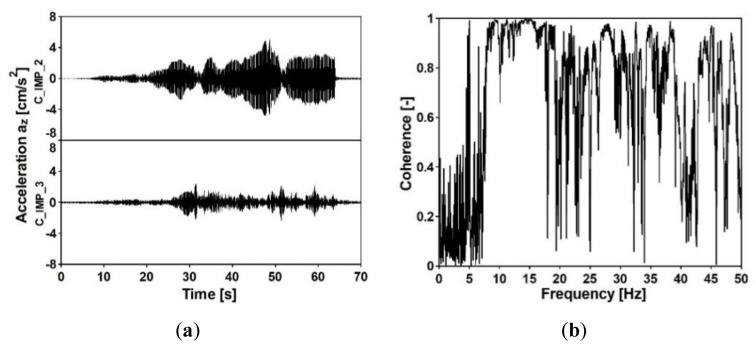
(**a**) Acceleration-time histories at input measurement points C_IMP_21 and C_IMP_3 of the cable-stayed footbridge due to the exponential sweep and (**b**) coherence function of the signals at points C_IMP_2 and C_IMP_3.

**Figure 15:**
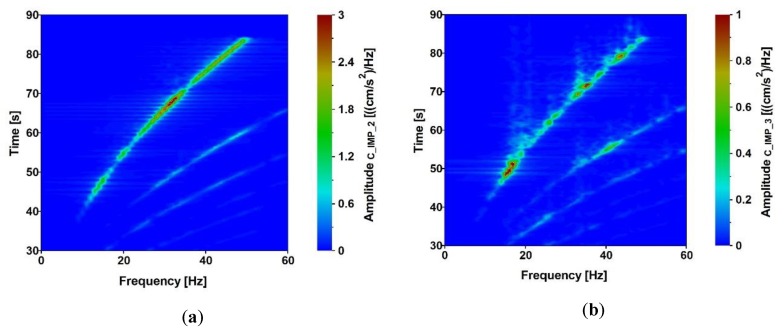
Time-frequency characteristics of the excitation at points (**a**) C_IMP_2 and (**b**) C_IMP_3 of the cable-stayed footbridge due to exponential sweep.

Acceleration-time histories in vertical direction, which occurred at the structures’ supports due to the exponential sweep, were registered at all input measurement points. The analyses of the signals are presented in Figure 12, Figure 13, Figure 14 and Figure 15. The signals registered at points F_IMP_1 and F_IMP_2 of the frame footbridge are shown in Figure 12a, whereas signals collected at points C_IMP_2 and C_IMP_3 of the cable-stayed footbridge are presented in Figure 14a. The coherence functions (CH) between the signals registered at points F_IMP_1 and F_IMP_2 as well as at points C_IMP_2 and C_IMP_3 were calculated and shown in Figure 12b and Figure 14b. This allowed for the recognition of the incoherence effect that may occur due to the SVEGM phenomenon. Next, the time-frequency characteristics of the examined nonuniform kinematic excitation are shown in Figure 13 and Figure 15 for both footbridges, respectively. 

In Figure 12b and Figure 14b, a high level of noise can be observed. This random noise could be generated by activities in the environment where data acquisition was carried out. It could be created by traffic, like truck and vehicles moving nearby, wind or a river (in case of the experiment on the cable-stayed footbridge). The noise could also originate from refraction and reflection of waves generated by the vibroseis and it was detected by the receivers with the signal. As the noise was not suppressed or removed from the signals registered at the supports of the footbridges, the presented coherence functions between these signals are affected by a relatively high level of noise as well.

It can be observed (see Figure 12b) that the coherence between signals at point F_IMP_1 and F_IMP_2 of the frame footbridge is satisfactory. Especially, in the frequency range of 5 to 25 Hz, the values are located over 0.8. In the case of the cable-stayed footbridge the coherence between signals at point C_IMP_2 and C_IMP_3 is a bit worse (see Figure 14b). However, still, in the frequency range of 7 to 17 Hz the values are over 0.8. The weak coherence for frequencies less than 5 Hz resulted from the fact, that, due to physical limitations, the ground force output from the vibroseis at low frequencies is limited. In consequence, harmonic distortion at low frequencies appears that results in significant loss of coherence. However, the investigation of the coherence functions proved that for seismic signals with dominant frequencies located in the range of 5 to 15 Hz, the adopted model of nonuniform kinematic excitation in which the loss of coherence is neglected can be adequate. The time-frequency characteristics of the excitation, shown in Figure 13 and Figure 15 for both footbridges, respectively, illustrate the intensity of the obtained signals and the time period of low frequencies registered at the footbridges’ supports. It can be observed that the signals on the consecutive structures’ supports are similar as far as the frequency content is considered.

**Figure 16:**
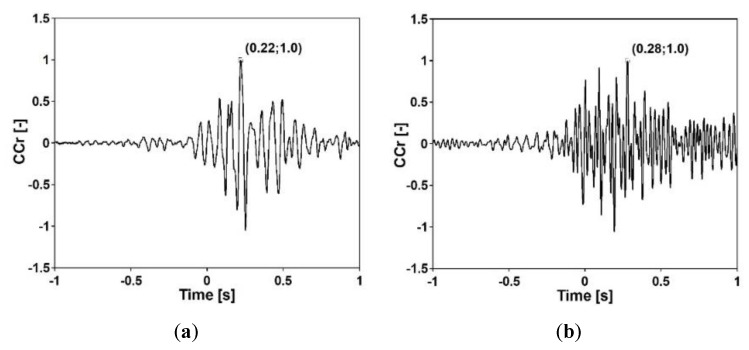
Cross-correlation functions between signals caused by exponential sweep at points: (**a**) F_IMP_1 and F_IMP_2 of the frame and (**b**) C_IMP_2 and C_IMP_3 of the cable-stayed footbridge.

The apparent wave velocity was evaluated on the basis of the cross-correlation functions (CCr) between the acceleration-time histories registered at two consecutive structure’s supports for both footbridges. The CCr functions (Equation (11)) between the signals at points F_IMP_1 and F_IMP_2 (see Figure 12a) of the frame footbridge and between the signals at points C_IMP_2 and C_IMP_3 (see Figure 14a) of the cable-stayed footbridge were calculated. The obtained estimators are presented in Figure 16. The maximum values of the CCr functions indicated the time shifts of signals registered on two consecutive structures’ supports. The apparent wave velocity was calculated as a quotient of the distance between the input measurement points (44.75 m between F_IMP_1 and F_IMP_2 and 60.00 m between C_IMP_2 and C_IMP_3) and the value of time shifts of the waves (0.22 and 0.28 s for the frame and cable-stayed footbridge, respectively). For the frame footbridge the apparent wave velocity was 203 m/s, whereas for the cable-stayed footbridge 214 m/s. 

**Figure 17:**
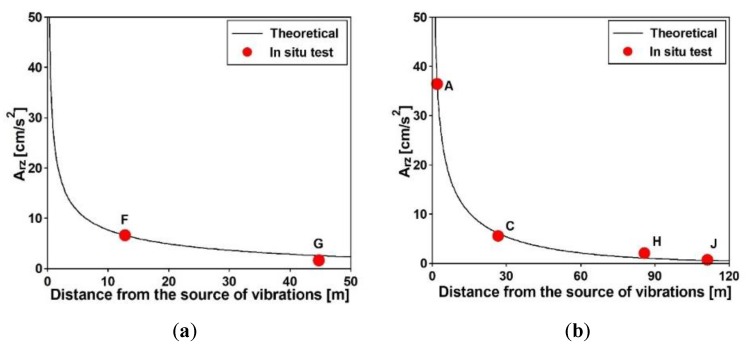
Comparison of the maximum accelerations, registered during the exponential sweeps (**a**) at two input points of the frame footbridge and (**b**) at four input points of the cable-stayed footbridge with the artificial curves of amplitude reductions.

The artificial kinematic excitation, i.e., acceleration-time histories applied to all supports of the structures, was generated based on the adopted model of nonuniform kinematic excitation. As a basic acceleration-time history was applied for the first supports of the structures at the input points (F_IMP_1 and C_IMP_1), the registered excitation resulting from the exponential sweeps were used. For the generation of excitations of the consecutive supports at the points (F_IMP_2 and C_IMP_2, C_IMP_3, C_IMP_4, for both footbridge, respectively) the apparent wave velocities of 203 and 214 m/s, were used for the frame and cable-stayed footbridges, respectively. The decrease of vibration amplitudes, caused by a distance from the source of vibration, was evaluated from Equation (13). The maximum accelerations, registered during the sweeps at two input points of the frame footbridge (F_IMP) and at four input points of the cable-stayed footbridge (C_IMP), were compared with the artificial curves of amplitude reductions in Figure 17. It may be stated that the maximum measured values of accelerations are in a very good agreement with their theoretical prediction. 

**Figure 18:**
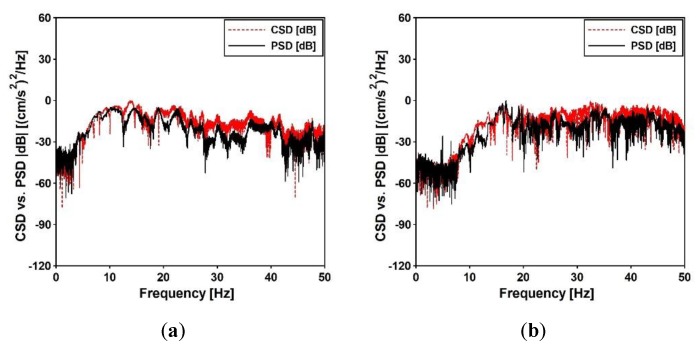
PSD function of registered acceleration-time history due to the exponential sweep vs. CSD function between registered and artificial acceleration-time histories for (**a**) point F_IMP_2 of the frame footbridge and (**b**) point C_IMP_3 of the cable-stayed footbridge.

The validation of the theoretical model of nonuniform kinematic excitation through the similarity assessment of the measured and artificial excitations was performed using the power spectral density (PSD) and cross-spectral density (CSD), which were calculated at all supports of the footbridges. The PSD and CSD functions calculated for the input points F_IMP_2 and C_IMP_3 of both footbridges are compared in Figure 18. Again, a good agreement of both functions was observed which confirmed the similarity of the registered and the artificial acceleration-time histories. The high level of noise in PSD functions, visible in Figure 18, was generated by activities in the environment where data acquisition was performed, as explained previously for the coherence functions (Figure 12b and Figure 14b). 

Based on the above investigation it may be concluded that the adopted theoretical model of nonuniform kinematic excitation was positively validated with regard to the experimentally detected nonuniform kinematic excitation.

### 7.3. Stage 3: Experimental vs. Numerical Dynamic Performance of Footbridges under Known Nonuniform Kinematic Excitation

The experimental dynamic responses of the footbridges were obtained from loading generated by the vibroseis at a constant frequency (20 Hz) with 10 s duration sweeps. This loading was acting on the footbridges’ supports as nonuniform kinematic excitation. The accelerations at all input (F_IMP and C_IMP) and output points (F_OMP and C_OMP) were measured. Therefore, the input data, the accelerations acting on the footbridges supports and measured at the input points, the output data, and the dynamic responses of footbridges in terms of the accelerations measured at the output points were experimentally measured. 

The dynamic responses of the footbridges were numerically calculated. The acceleration-time histories, which were registered in the input points (F_IMP and C_IPM) were applied as kinematic excitations of both footbridges supports in the FE analysis. The acceleration time histories calculated at the output points (F_OMP and C_OMP) were the numerical dynamic responses of the footbridges. 

The dynamic responses of the footbridges under the nonuniform kinematic excitation were calculated using full-time history analysis. It was conducted with the Hilber–Hughes–Taylor time integration algorithm provided in the ABAQUS/Standard software for a direct step-by-step solution [27]. The step varied from 10 to 5s, and from 10 to 2 s, according to convergence requirements. 

The Rayleigh model of damping with coefficients α (for mass proportional damping) and β (for stiffness proportional damping) was used for the numerical simulations. Damping ratio values were adopted from the experimental modal model (see Table 1). The following values of coefficients were applied: α=0.4003, β=0.0005 for the frame footbridge (obtained for the 1st and 2nd natural frequency, see Table 1) and α=0.3075, β=0.0003 for the cable-stayed footbridge (for the 1st and 3rd natural frequency, see Table 1).

The validation of numerical performance of footbridges and the appropriateness of the FE procedures used for dynamic analyses was done by comparison of the numerical and experimental accelerations collected at output measurement points F_OMP and C_OMP (Table 2 and Table 3). The mean errors between the maximal experimental and numerical accelerations were 11 and 20% for the frame and the cable-stayed footbridge, respectively. Taking into consideration the complexity of the FE models as well as the nature of SVEGM, it can be stated that the measured and calculated accelerations are in sufficient agreement and the numerical performance of footbridges strongly resembles the experimentally found behavior of the structures. 

Due to the complex experimental testing, carried out on three stages, the results of further numerical seismic analyses reflect the real dynamic performance of the structures under nonuniform ground motions with high similarity.

## 8. Dynamic Performance of the Footbridges under a Selected Seismic Shock—Practical Application of the Measurement Results of the SVEGM Effect

### 8.1. Seismic Event Chosen for Calculations

In the numerical part of the research, the seismic dynamic responses of the structures to a selected seismic shock were calculated. Both the uniform and nonuniform models of kinematic excitation were applied for the dynamic calculations that enabled to demonstrate and quantify the influence of SVEGM on the seismic assessment of the footbridges. 

**Figure 19:**
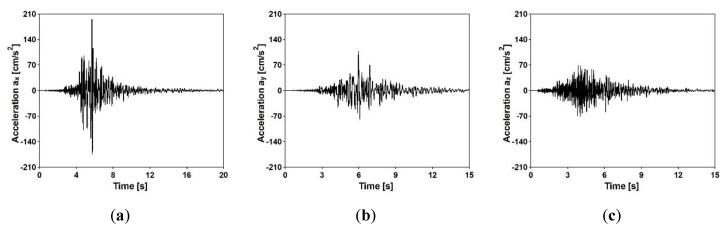
Acceleration time-histories of the seismic event in (**a**) X direction, (**b**) Y direction, and (**c**) Z direction [31].

**Figure 20:**
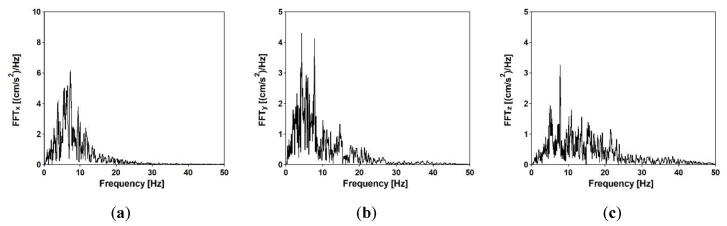
Frequency spectra of accelerations for the seismic event in (**a**) X direction, (**b**) Y direction, and (**c**) Z direction.

An earthquake of the Richter magnitude 5.1 was used as kinematic excitation of the footbridges in the time history analyses (THA) [31]. The recorded acceleration-time histories of the shock are presented in Figure 19, whereas the frequency spectra are shown in Figure 20. It can be observed that the dominant frequencies of the shock are located in the range of 5 to 8 Hz.

This seismic event was chosen as the 2012 Northern Italy Earthquakes represent a good case study regarding the losses caused by a moderate earthquake in a densely populated and economically well-developed area in European Union [32]. Moreover, the experimental investigation of the coherence functions (see Figure 12b and Figure 14b) proved that the validated model of nonuniform kinematic excitation, in which the loss of coherence is neglected, is appropriate for excitations with the dominant frequency range around 5–15 Hz. 

**Figure 21:**
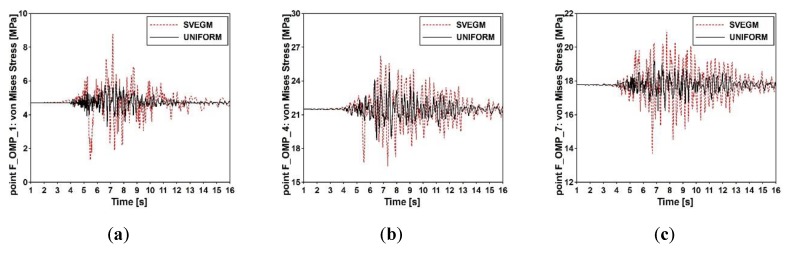
Von Mises stress-time histories for the frame footbridge for output control points: (**a**) F_OMP_1, (**b**) F_OMP_4, and (**c**) F_OMP_7.

The accelerations presented in Figure 21 were applied to all supports of the structures as the uniform kinematic excitation. For the case of the nonuniform excitation, the registered seismic shock was used as the excitation of the first support of the footbridges. The artificial acceleration-time histories of excitations of the next supports of the structures were obtained based on this loading. The wave velocities of 203 and 214 m/s, measured during in situ experiments for both footbridges, respectively, were assumed (see Figure 16).

### 8.2. Dynamic Performance of the Single-Span Steel Frame Footbridge 

The dynamic performance of the structures was assessed using the calculated von Mises stresses. The von Mises stress-time histories for the output points F_OMP_1, F_OMP_4, and F_OMP_7 located on the left frame of the footbridge (see Figure 4a) are presented in Figure 21. The stress-time histories were obtained for two variants, i.e., the uniform and the nonuniform (with the seismic wave velocity 203 m/s) excitation. The comparison of stress-time histories obtained for all output points demonstrate that the dynamic response of the footbridge to the shock is greater when the model of nonuniform excitation is applied. 

**Figure 22:**
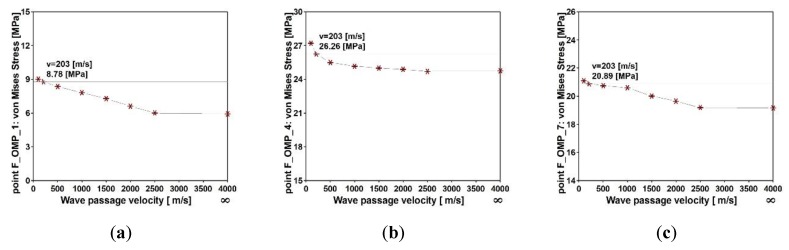
Maximum von Mises stresses for different values of wave velocity for the frame footbridge at output points: (**a**) F_OMP_1, (**b**) F_OMP_4, and (**c**) F_OMP_7.

**Figure 23:**
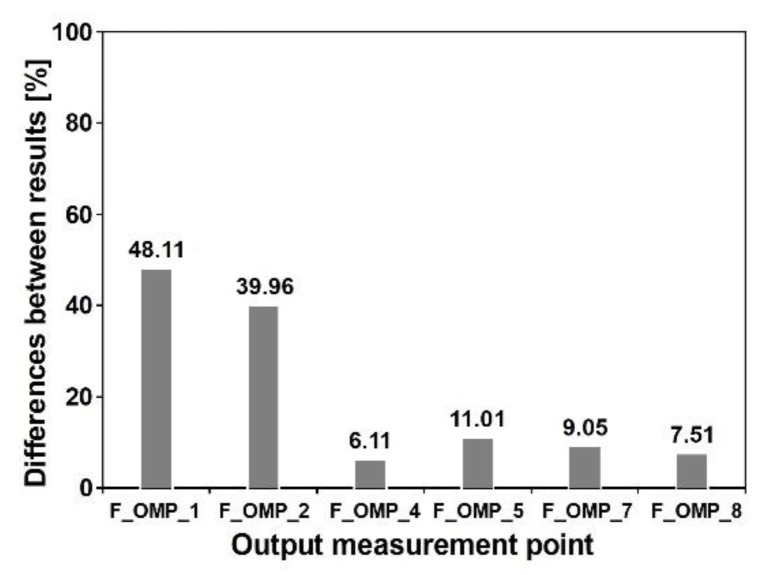
Differences between Mises stresses obtained for uniform and nonuniform model of kinematic excitation with wave velocity 203 m/s at output points of the frame footbridge.

The multi-variants analysis was conducted next to assess the influence of wave velocity on the structural response. The maximum values of von Mises stresses obtained at the output points F_OMP_1, F_OMP_4 and F_OMP_7 for different wave velocities (100, 500, 1000, 1500, 2000, and 250 m/s) are presented in Figure 22. The maximum values were obtained when nonuniform model of excitation was applied and the seismic wave velocity was 100 m/s. For the model of uniform seismic excitation, the obtained values of Mises stresses were the lowest. The differences between results at all output points were in the range of 10 to 50% (see Figure 23). In summary, the most significant influence of SVEGM was observed at the output points F_OMP_1 and F_OMP_2. These points were localized closely to the stiff nodes of the steel frame (see Figure 4a). The less significant differences were observed at output points F_OMP_4, F_OMP_5, F_OMP_7 and F_OMP_8 located in the middle of the upper beam of the frame.

### 8.3. Dynamic Performance of the Three-Span Cable-Stayed Footbridge

**Figure 24:**
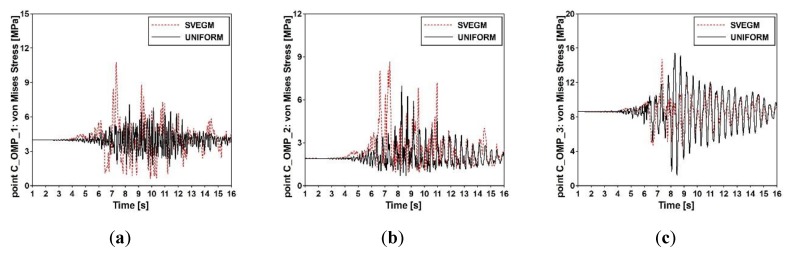
Von Mises stress-time histories for the cable-stayed footbridge at control points: (**a**) C_OMP_1, (**b**) C_OMP_2, and (**c**) C_OMP_3.

The von Mises stress-time histories obtained for the uniform and the nonuniform excitation (with the seismic wave velocity 214 m/s) at output point C_OMP_1 located on one extreme span and at output points C_OMP_2, C_OMP_3 located in the midspan of the cable-stayed footbridge (see Figure 5a) are presented in Figure 24.

The comparison of stress-time histories at all output points demonstrate that the dynamic response of the footbridge to the shock is greater at output points C_OMP_1 and C_OMP_2 for the model of nonuniform excitation. However, at output points C_OMP_3 a reverse situation is observable.

**Figure 25:**
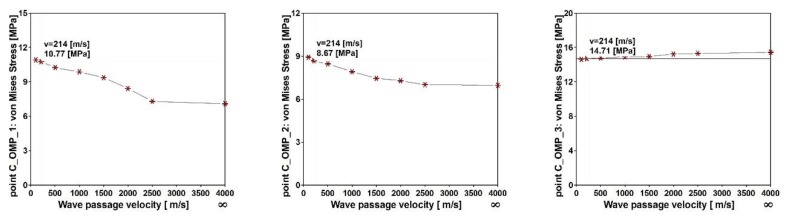
Maximum von Mises stresses for different values of wave passage velocity for the cable-stayed footbridge at output points: (**a**) C_OMP_1, (**b**) C_OMP_2, and (**c**) C_OMP_3.

**Figure 26:**
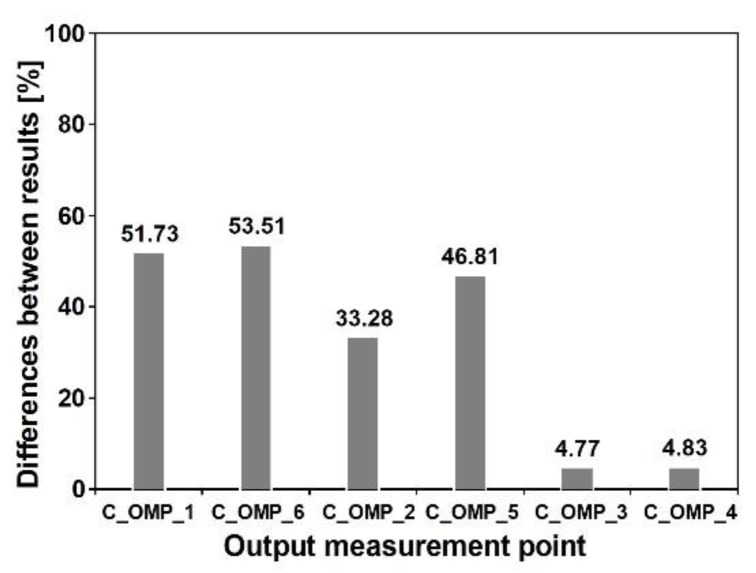
Differences between Mises stresses obtained for uniform and nonuniform model of kinematic excitation with wave velocity 214 m/s at output points of the cable-stayed footbridge.

For the cable-stayed footbridge, the multi-variants analysis was also conducted. The maximum von Mises stresses at output points C_OMP_1, C_OMP_2, and C_OMP_3 for different wave velocities are shown in Figure 25. For this footbridge, the differences between results are in the range of 5 to 50% (see Figure 26). In summary, for the cable-stayed footbridge two different scenarios at all output points were observed. For the output points C_OMP_1 and C_OPM_6 located in the left and right spans as well as for the C_OMP_2 and C_OMP_5 located in the midspan closely to the pillars (see Figure 5a) the maximum values of von Mises stresses occurred for the model of nonuniform kinematic excitation with the lowest wave velocity of 100 m/s. For the output points C_OMP_3 and C_OPM_4, located in the midspan, the numerical simulations with the model of uniform excitation provided more conservative results than the model of nonuniform excitation.

## 9. Conclusions

The first experimental part of the research served for the validation of the FE models of structures, the theoretical model of nonuniform excitation and the appropriateness of the FE procedures used for dynamic analyses. The following conclusions can be formulated on the basis of the experimental testing part:

1. The numerical modal models of both footbridges, obtained through assembling the FE models of footbridges and the through the calculation of the dynamic properties, were positively validated with regard to the experimental modal models obtained through the operational modal analysis (OMA) techniques. The differences between the experimental and numerical natural frequencies were generally less than 10%. The comparison of the experimental and numerical mode shapes also revealed a good agreement between them since the values of MAC indices were satisfactory for both structures.
2. The non-destructive test with the vibroseis generating kinematic excitation was carried out. On the basis of the signals collected at all footbridges’ supports, nonuniform kinematic excitation was detected. The apparent wave velocities, obtained on the basis of cross-correlation functions between the acceleration-time histories at the consecutive supports of structures, are representative for the clayey subsoils of both the frame and the cable-stayed footbridge. Also, the coherence functions between these signals were extracted. The similarity and the frequency consistency of the data recorded at the input points was satisfactory, especially for the frequency range of 5 to 15 Hz. Therefore, for seismic signals with such dominant frequency range, the adopted model of nonuniform kinematic excitation, in which the loss of coherence is neglected, can be appropriate.
3. The artificial kinematic excitation was generated on the basis of the adopted model of nonuniform kinematic excitation. The obtained PSD functions of the acceleration-time histories registered at all supports as well as the CSD functions between the registered and artificial acceleration-time histories for all supports confirmed the strong similarity of the measured and numerical signals. Therefore, the adopted model of nonuniform kinematic excitation was positively validated with regard to the experimentally detected nonuniform excitation.
4. The experimental and numerical assessments of the footbridges performance under the known dynamic excitation induced by the vibroseis were carried out. As the overall agreement is satisfactory, the FE models and procedures were positively validated by linking full-scale experiments and numerical calculations. Therefore, the numerical performance of structures reflects the real dynamic performance under known nonuniform excitation.

In the second part of the research the numerical seismic assessments of the footbridges subjected to the representative seismic earthquake were presented. The following conclusions can be formulated on the basis of the numerical part.

1. For the stiff and relatively short single-span steel frame footbridge, the maximum structural response was obtained for the model of nonuniform kinematic excitation with the lowest seismic wave velocity. For the model of uniform seismic excitation, the response was the weakest. The most significant influence of the SVEGM effect was observed when the points were localized closely to the stiff node of the steel frame. The less significant differences were observed at points located in the middle of the upper beam of the frame. Therefore, in case of stiff frame footbridges the pseudo-static effects resulting from the nonuniformity of excitation enhance the dynamic response of a structure, especially disturbing zones located closely to stiff supports.
2. For the flexible cable-stayed footbridge, two different scenarios were observed. For the output points located in the extreme spans, as well as in the midspan closely to the pillars, the maximum response occurred for the model of nonuniform kinematic excitation with the lowest wave velocity. For the output points located in the middle of the main span, the numerical simulations with the model of uniform excitation provided more conservative results. Therefore, in case of flexible cable-stayed footbridges the pseudo-static effects originated from SVEGM disturb zones located closely to the structure supports, whereas in the midspan of the structure the inertial effects caused by the uniform excitation are stronger.
3. It is reasonable to consider the SVEGM effect for the seismic assessments of footbridges since this phenomenon may generate non-conservative results in comparison with results of classic analysis in which uniform seismic excitation is assumed.

Generally, the results of numerical seismic assessment of footbridges under nonuniform ground motion are reliable as they are based on the comprehensive experimental testing validating the implemented FE models and procedures. Therefore, the results of the whole research, both the experimental and the numerical part, might be applicable for the seismic assessment of the types of structures considered in the investigation.

The calculations were performed for the seismic event representative for Central Europe; however, more calculations based on some benchmark earthquakes, e.g., El-Centro or Kobe, are planned in future research to validate the proposed approach.

It should be emphasized that, in addition to the cognitive values of the research, the results presented in the paper may find practical application in the dynamic diagnostics of footbridges. They may also provide additional information for the industry, useful in the process of designing new objects erected in seismic areas.

## Figures and Tables

**Table 1:** Dynamic characteristics of the footbridges [6,7].

**The Single-Span Steel frame Footbridge—Bridge One**					
**Mode**	**Natural Frequency f_i_ [Hz]**		**Differences [%]**	**MAC_ii_ [–]**	**Logarithmic Decrement $\delta_{i}$ [–]**
	**FE Analysis**	**OMA**			
1	1.71	1.76	2.92	0.92	0.131
2	2.45	2.68	9.39	0.84	0.101
3	3.33	3.15	5.41	0.80	0.056
4	3.41	4.19	22.87	0.78	0.035
5	4.40	4.47	1.59	0.83	0.025
**The Three-Span Cable-Stayed Footbridge—Bridge Two**					
**Mode**	**Natural Frequency f_i_ [Hz]**		**Differences [%]**	**MAC_ii_ [–]**	**Logarithmic Decrement $\delta_{i}$ [–]**
	**FE Analysis**	**OMA**			
1	1.93	1.95	1.04	0.95	0.092
2	2.21	2.41	9.05	0.94	0.079
3	2.61	2.58	1.15	0.94	0.077
4	3.86	3.69	4.40	0.91	0.075
5	4.37	4.28	2.06	0.72	0.033

**Table 2:** The values of maximum acceleration in the output measurement points for the frame footbridge.

Method	Acceleration [cm/s^2^]								
	Axis	Output Measurement Point (F_OMP)							
		1	2	3	4	5	6	7	8
In situ experiment	X	5.98	6.80	3.52	3.02	4.39	5.77	4.48	3.67
	Y	3.49	2.75	4.05	3.29	6.90	5.23	6.91	5.69
	Z	12.49	9.14	21.08	6.88	9.82	5.67	4.89	4.79
FE analysis	X	5.38	5.91	3.82	3.39	4.89	6.06	4.98	4.07
	Y	3.91	3.04	3.93	3.15	7.54	5.38	6.31	5.97
	Z	12.32	11.59	18.37	7.94	8.27	6.91	5.59	5.54

**Table 3:** The values of maximum acceleration in the output measurement points for the cable-stayed footbridge.

Method	Acceleration [cm/s^2^]						
	Direction	Output Measurement Point (C_OMP)					
		1	2	3	4	5	6
In situ experiment	X	7.14	8.04	6.81	6.17	9.35	5.74
	Y	7.01	8.48	6.37	6.67	6.90	8.47
	Z	23.74	17.72	31.12	28.37	24.80	28.18
FE analysis	X	8.23	9.87	7.55	7.49	11.42	7.38
	Y	8.99	10.52	7.79	7.21	8.51	11.37
	Z	29.72	21.72	35.31	36.25	28.37	29.73
